# A rapid, non-invasive method for fatigue detection based on voice information

**DOI:** 10.3389/fcell.2022.994001

**Published:** 2022-09-13

**Authors:** Xiujie Gao, Kefeng Ma, Honglian Yang, Kun Wang, Bo Fu, Yingwen Zhu, Xiaojun She, Bo Cui

**Affiliations:** Tianjin Institute of Environmental and Operational Medicine, Tianjin, China

**Keywords:** fatigue detection, speech features, acoustic biomarkers, vocal print, fatigue scale

## Abstract

Fatigue results from a series of physiological and psychological changes due to continuous energy consumption. It can affect the physiological states of operators, thereby reducing their labor capacity. Fatigue can also reduce efficiency and, in serious cases, cause severe accidents. In addition, it can trigger pathological-related changes. By establishing appropriate methods to closely monitor the fatigue status of personnel and relieve the fatigue on time, operation-related injuries can be reduced. Existing fatigue detection methods mostly include subjective methods, such as fatigue scales, or those involving the use of professional instruments, which are more demanding for operators and cannot detect fatigue levels in real time. Speech contains information that can be used as acoustic biomarkers to monitor physiological and psychological statuses. In this study, we constructed a fatigue model based on the method of sleep deprivation by collecting various physiological indexes, such as P300 and glucocorticoid level in saliva, as well as fatigue questionnaires filled by 15 participants under different fatigue procedures and graded the fatigue levels accordingly. We then extracted the speech features at different instances and constructed a model to match the speech features and the degree of fatigue using a machine learning algorithm. Thus, we established a method to rapidly judge the degree of fatigue based on speech. The accuracy of the judgment based on unitary voice could reach 94%, whereas that based on long speech could reach 81%. Our fatigue detection method based on acoustic information can easily and rapidly determine the fatigue levels of the participants. This method can operate in real time and is non-invasive and efficient. Moreover, it can be combined with the advantages of information technology and big data to expand its applicability.

## 1 Introduction

Fatigue is generally used to describe physical and/or mental weariness, which extends beyond normal tiredness and is closely related to sleep. Fatigue is usually divided into three types: transient fatigue caused by extreme sleep restriction or extended hours awake within 1 or 2 days, cumulative fatigue caused by repeated mild sleep restriction or extended hours awake across a series of days, and circadian fatigue, that is, the reduced performance during nighttime hours (Lock et al., 2018). A greater understanding of fatigue may improve both research and clinical care.

Fatigue could negatively impact work performance, family life, and social relationships (Rosenthal et al., 2008; Caldwell et al., 2019). It leads to a series of physiological and psychological changes due to continuous energy consumption. Fatigue can affect the physiological states of operators, thereby reducing their labor capacity. Notably, changes in physiological aspects can have significant implications on sensory and motor metabolic functions, which could cause significant decreases in decision-making, problem-solving, and psychomotor skills, vigilance, processing speed, and working and long-term memory after sleep deprivation (Lim and Dinges, 2010). This may lead to work disorders due to an increase in ineffective decisions or the likelihood of error. Therefore, fatigue can reduce efficiency and, in serious cases, contribute to a meaningful proportion of operational accidents and incidents (Uehli et al., 2014; Arsintescu et al., 2019). In addition, it can trigger pathological-related changes in multiple organs. For example, it has been reported that prolonged fatigued working conditions can trigger pathological-related issues such as skeletal muscle damage (Constantin-Teodosiu and Constantin, 2021), hearing damage lesions such as sudden deafness (Zhou et al., 2020; Fu et al., 2021; Holman et al., 2021; Fu et al., 2022), gastrointestinal complications, and cardiovascular issues (Natelson et al., 2021). By establishing appropriate methods to closely monitor the fatigue status of personnel and relieve the fatigue on time, operation-related injuries can be reduced.

The human voice is produced by the vibration of the vocal cords. Each voice has its unique characteristics depending on the size of the vocal cavity, including the throat, nasal cavity, and oral cavity. The shapes, sizes, and positions of these organs determine the tension in the vocal cords and ranges of the sound frequencies produced (Zaske et al., 2017). Normal growth and aging can affect the histopathological changes in the vocal folds. However, some changes are caused by abnormal physiological states, such as emotional changes, fatigue, or diseases (Kuhn, 2014). For instance, a change in the vocal cords following COVID-19 infection has been reported (Jungbauer et al., 2021). Owing to the unique feature of each voice, voiceprint recognition has been widely used in many fields (Sun et al., 2018). Several legal cases have used voiceprints for speaker identification under certain environmental characteristics. The FBI conducted statistics on 2,000 cases related to voiceprints, and the error rate was only 0.31% (Koenig, 1986). Many algorithms have been reported in the field of voiceprint recognition to extract and recognize voiceprint features (Sun et al., 2018), and there are also many new achievements in the research and development of wearable devices. Wearable active sensors have extensive applications in mobile biosensing and human-machine interaction (Li et al., 2017; Adao Martins et al., 2021).

By analyzing the characteristic parameters in speech, we can identify and classify certain diseases for diagnostic, prognostic, and preventive measures (Latinus and Belin, 2011; Petkov and Vuong, 2013). Speech-based methods could be more accurate, faster, simpler, and cheaper than traditional methods. The eigenvalues in the vocal information in human speech are closely related to health conditions. For example, patients suffering from neurodegenerative, cardiovascular, and mental disorders may experience vocal changes (Ahmed et al., 2013; Giddens et al., 2013; Kunin et al., 2020; Yang et al., 2020). In addition, the eigenvalues may reflect fatigue levels. The detection of fatigue levels has become an important research topic in the field of brain cognition (Penner et al., 2009). At present, brain fatigue detection is classified into subjective and objective detections. Objective detection methods primarily use physiological signals, such as electroencephalogram (EEG) and electromyography (EMG), but the conventional EEG and EMG methods are inconvenient to operate in the detection process and need to interrupt the normal operation process of the participant (Furutera et al., 2021). The physiological signals are susceptible to several other factors, such as environmental conditions, emotions, and pathophysiological issues (Adao Martins et al., 2021). Other detection methods use physiological signals or changes in behavioral characteristics, such as eye movements (Sampei et al., 2016; Ko et al., 2020), but these signals are not easily detected either. The existing methods are demanding for operators, time-consuming, and cannot detect the fatigue level in real time.

In this experiment, we established a fatigue model by sleep deprivation and collected the voice information of participants in different fatigue states. By developing a voice feature-extraction software, we extracted the features in the voice information. Subsequently, we explored the matching relationship between the voice information features and the fatigue levels by combining the results of the fatigue questionnaires and relevant physiological indicators of the participants through a machine learning method. Using these results, we developed a method to monitor the level of physical fatigue using vocal information.

## 2 Materials and methods

### 2.1 Participants’ recruitment

In our experiments, 15 participants were enrolled. They were all male students with an age range of 23–25 years. All participants were in good health, had good lifestyle habits, no drug histories within the previous months, regular routines, and no recent fatigue-related habits such as staying up late. They were required to stay awake during the 36-h duration of the experiment. Further, they were asked to fill in the fatigue questionnaire, provide their saliva samples, and record audio files at several instances as per the experimental procedure.

The participants’ voice information at 0 h was used as the control group (non-fatigue group), and the data after 36 h of sleep deprivation was used as the fatigue group. In addition, the questionnaire and P300 test results were combined to verify and exclude abnormal data.

### 2.2 Experimental data collection

#### 2.2.1 Collection of questionnaires

The fatigue questionnaire used in this study was the Stanford Sleepiness Scale. It was filled out by the participants according to their subjective fatigue level every 12 h. The results of all 15 participants at different time points were used to determine the corresponding fatigue grades.

#### 2.2.2 Collection of saliva

The participants were forbidden from eating or drinking 1 h before the collection. They had to rinse their mouth 30 min before collection, insert cotton balls from the chewing saliva collection tubes into their mouth, chew them for approximately 1 min, and then put them back into the collection tubes. The volume of saliva collected was approximately in the range of 2–3 ml. The collection tubes were centrifuged in a freezing centrifuge to collect the saliva samples for subsequent cortisol detection.

#### 2.2.3 Cortisol detection

Cortisol detection in the saliva was conducted using a cortisol detection kit (RE52611, IBL International GmbH). The cortisol concentration was detected by an enzyme-linked immunosorbent assay method according to the manufacturer’s protocol. The absorbance of the samples at a wavelength of 450 nm was detected *via* a spectrophotometer, and the cortisol content was determined using the generated standard curve.

#### 2.2.4 Acquisition of the amplitude and delay of P300 using smart-EP-ASSR instrument

According to the instrument operation protocol, electrodes were attached to four points on the participants. The recording electrode was placed in the Cz position, the reference in the bilateral mastoid, and the ground in the Fz position. By using an oddball mode of the detecting system, the non-target stimulation was 1,000 Hz, 60 dB with 80% probability, and the target stimulation was 2,000 Hz, 70 dB with 20% probability. Target stimuli were superimposed 50 times. During the process, the participants were asked to memorize the times the target stimulus appeared and the times of acquisitions. The amplitude and delay data of P300 were recorded using the detecting system on the PC.

#### 2.2.5 Voice data acquisition

The participants read a short text audibly. Their voices were recorded using a voice recorder (TX650, Sony Corporation) and saved for subsequent analysis. To minimize noise interference, the recording was conducted in a relatively quiet, independent room. Each participant recorded six vowels, four daily phrases, and a 300-word paragraph at each time point.

### 2.3 Statistical analysis of fatigue questionnaire data

The data were statistically analyzed and are expressed as the mean ± standard error of mean. The *t*-test was used to detect significant differences, with *p* < 0.05 representing a significant difference.

### 2.4 Fatigue assessment based on speech and audio features

#### 2.4.1 Analysis of recorded audio files

For each audio file, 19 parameters were collected. The audio files were recorded into the speech analysis module, and the values of the 19 parameters were extracted for subsequent analysis and determination of fatigue status: the parameters include the fundamental frequency (F0), energy, zero-crossing (Zcr), harmonics-to-noise ratio (HNR), voice quality (Jitter, Shimmer), loudness, and 12 Mel-frequency Cepstral coefficients (MFCCs, 1–12).

#### 2.4.2 Classification of fatigue status

The results of the questionnaire, concentration level of glucocorticoid (GC), and parameters of P300 were analyzed. These three indicators were comprehensively considered. The participants were classified into two groups: fatigue and non-fatigue. The speech data of all participants at 0 h was set as the non-fatigue group. The participants’ data at 36 h of sleep deprivation, including the GC level, questionnaire, and P300 results, showed significant differences compared with those at the experimental starting point of 0 h and were set as the fatigue group.

#### 2.4.3 Fatigue assessment based on audio features

Audios of vowel sounds, phrases, and ordinary conversations were obtained at 32 kHz. From each frame, which was 20 ms long with 10 ms overlap, we extracted several types of audio features, such as the fundamental frequency, energy, and zero-crossing rate. The average audio features of the frames were used as input features of the fatigue level classifiers. By using P300 as ground truth, we trained several commonly used classifiers, including linear regression (LR) (Nguyen et al., 2021), linear discriminant analysis (LDA) (Dornaika and Khoder, 2020), K-nearest neighbor (KNN) (Abu Alfeilat et al., 2019), classification and regression trees (CART) (Johns et al., 2021), naive Bayes classifier (NB) (Sugahara and Ueno, 2021), support vector machine (SVM) (Huang et al., 2018), and multilayer perceptron (MLP) (Panghal and Kumar, 2021), to classify the fatigue level of each audio input. Leave-one-out (LOO) cross-validation (Luo et al., 2015) was used to guarantee the generalization performance of our models. During each cross-validation fold, we used the samples of one participant as the validation set and the remaining samples as the training set.

Particularly, for the hyperparameters of the SVM, we used different kernel functions, such as linear, radial basis function, polynomial, and sigmoid kernels. We found that the linear kernel outperformed the others. Therefore, the experiments were conducted via the SVM using a linear kernel.

## 3 Results

### 3.1 Statistics of participants’ information and schematic of the experimental procedure

Table 1 presents the basic information of the 15 participants. Notably, they gradually entered the fatigue state according to the aforementioned method, and the relevant indexes were tested at different time points.

**TABLE 1:** Characteristics of the participants.

Characteristic	Data
Numbers of participants	15
Sex	Male
Age (year)	23.5 ± 2.0
Bodyweight (kg)	69.4 ± 9.0
Health condition	Healthy

**FIGURE 1:**
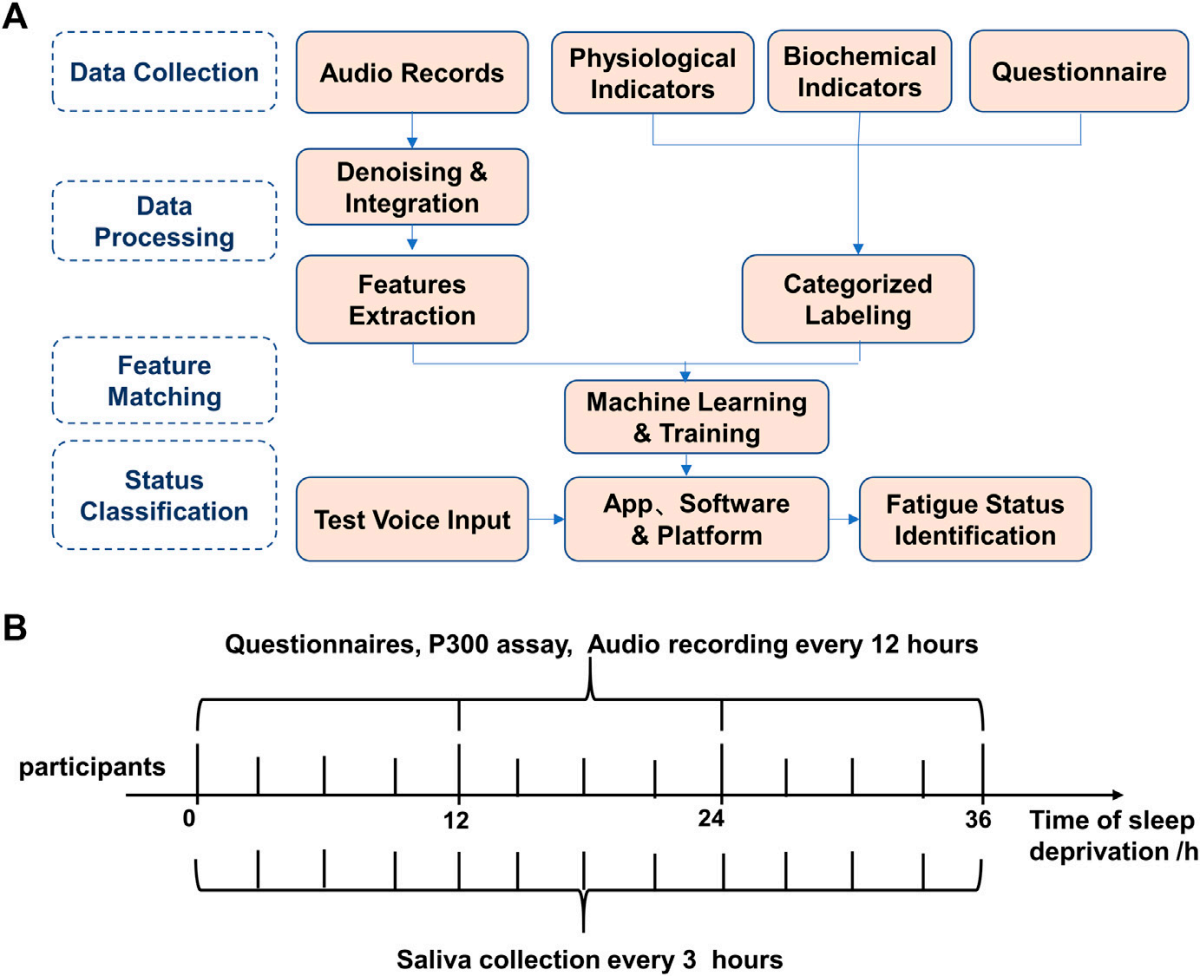
Overall experimental procedure of speech-based fatigue grading. **(A)** The overall experimental flow chart of the speech-based fatigue classification. **(B)** Experimental timeline, time points, and measures. Questionnaires, P300 and audio data were collected every 12 h, and saliva was collected every 3 h and audio data were collected every 12 h, and saliva was collected every 3 h.

The flow chart of the experiment is shown in Figure 1A. The experimental timeline, time points, and measures are shown in Figure 1B. The questionnaire, P300 assay, saliva collection, and audio recording on each time point were carried out accordingly.

### 3.2 Results of biochemical data and electrophysiological indexes detected in participants at different fatigue levels

**FIGURE 2:**
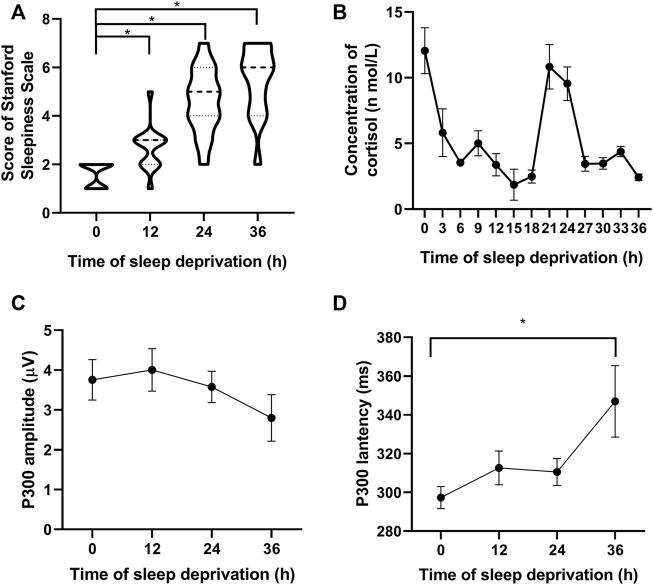
Participants’ fatigue gradually increased with an increase in sleep deprivation time. **(A)** Subjective fatigue scale score of the participants. **(B)** Salivary cortisol level varies according to a 24-h rhythm. **(C)** Amplitude of P300 decreases over time, and **(D)** Latency of P300 gradually increases with time. *p < 0.05 vs. starting points.

The results showed a gradual increase in the fatigue level of the participants over time, as shown in Figure 2A. The participants’ salivary cortisol levels varied according to a 24-h rhythm: decreased after 12 h, rebounded after 24 h, and decreased again after 36 h. In the comparison, the salivary cortisol levels tended to decrease with increasing fatigue at the same rhythmical point (Figure 2B). The amplitude of the P300 slightly increased after 12 h and then gradually decreased after 24 and 36 h (Figure 2C). Compared with that at the starting points, the latency of P300 after 36 h showed a significant increase (Figure 2D).

The three indicators, namely P300, the fatigue questionnaire, and the sleep deprivation time, were combined to classify and label the participants’ fatigue levels. A matching relationship was established between voice messages and fatigue levels (Li and Mills, 2019), and each voice message was tagged with a fatigue classification label (fatigue or non-fatigue).

### 3.3 Using SVM method to judge fatigue level according to single vowel information

**FIGURE 3:**
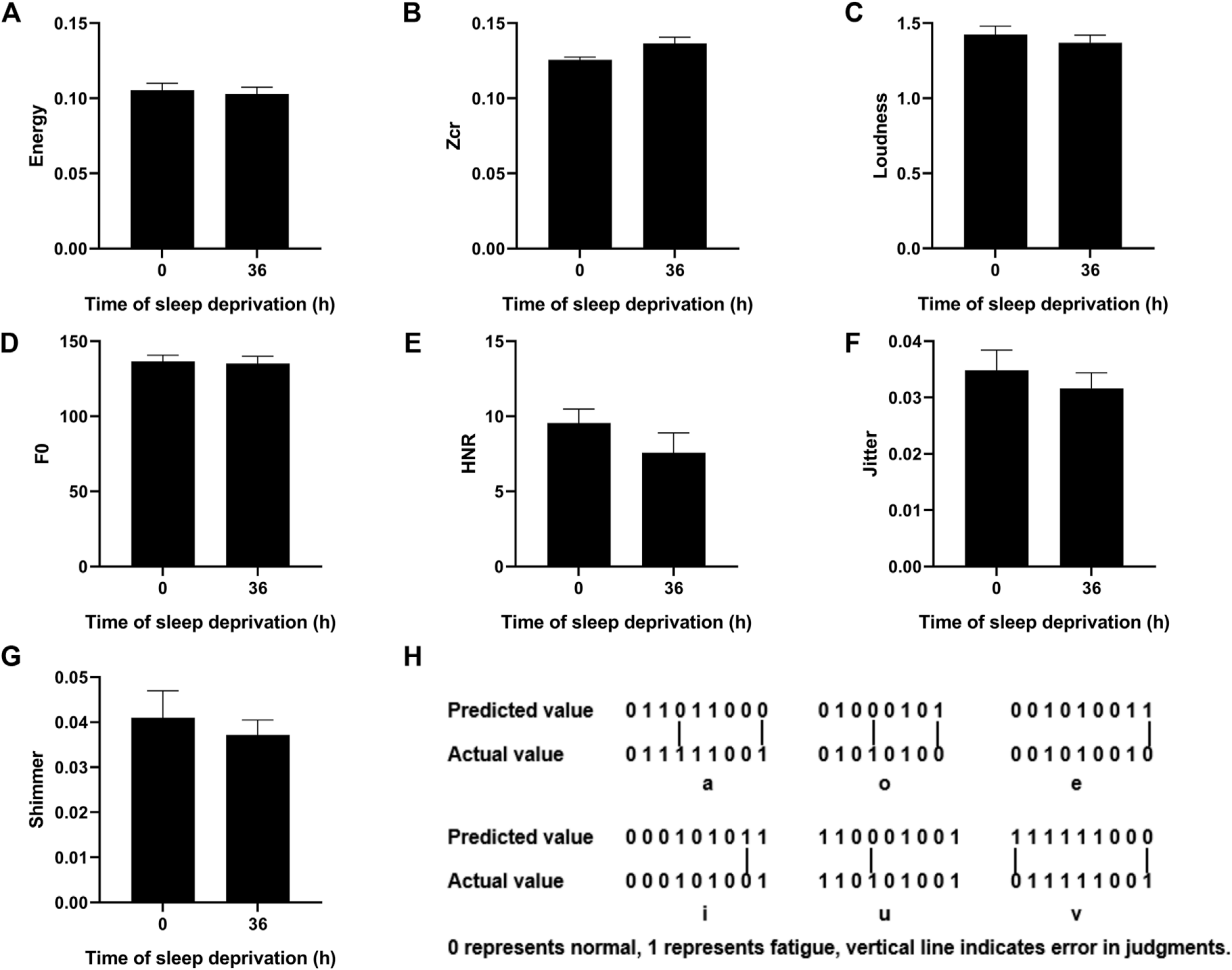
Changes in phonetic feature parameters of the vowel “a” after 36 h sleep deprivation and comparison of predicted and actual values of different vowels. **(A–G)** Value of the Energy/Zcr/Loudness/F0/HNR/Jitter/Shimmer for the vowel “a” before and after 36 h of sleep deprivation. **(H)** Fatigue prediction by the SVM method.

We calculated the values of energy, zcr, loudness, F0, HNR, Jitter, and Shimmer for the vowel “a” after 36 h of sleep deprivation and found no significant differences compared with those at the onset point (Figures 3A–G). Then, we used the SVM model to make predictions and compare them with the labeled fatigue levels, as shown in Figure 3H. The number 0 represents normal (non-fatigue), and 1 represents fatigue. The predicted value is the result predicted by the SVM algorithm, and the actual value is the result of the judgment on whether the participant is fatigued as per the three parameters above. If the two parameters are consistent (both are 0 or 1), it means the prediction is accurate; otherwise, it means the prediction is inaccurate. The accuracy of the integrated six-vowels judgment is shown in Table 2. The results showed that based on the pure vowels recorded by the participants, the prediction accuracy could reach approximately 88% for single vowels and up to 94% for multi-vowels compared with that of the fatigue classification based on physiological parameters.

**TABLE 2:** Accuracies of different vowel classifications in the SVM model.

	Vowels used for prediction						
Vowels	a	o	e	i	u	v	Total
Accuracy	0.77	0.75	0.88	0.88	0.77	0.77	0.94

### 3.4 Fatigue classification based on speech information using a variety of neural network algorithms

**FIGURE 4:**
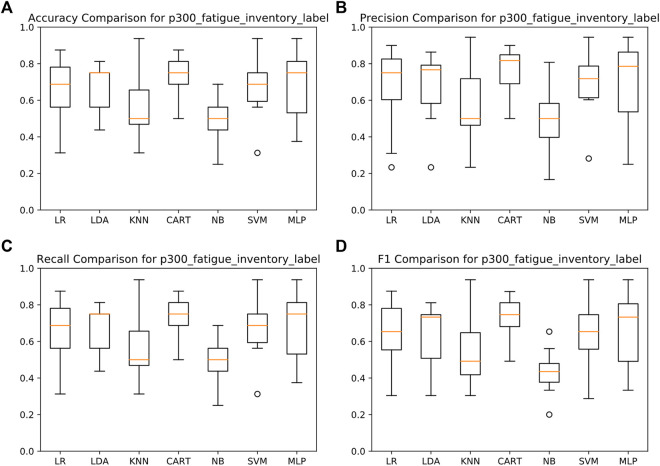
Detection results of fatigue assessment based on voice audio features. **(A)** Accuracy comparison for P300_faigue_inventory_label. **(B)** Precision comparison for P300_fatigue_inventory_label. **(C)** Recall comparison for p300_fatigue_inventory_label. **(D)** F1 comparison for P300_fatigue_inventory_label.

We analyzed and judged segmented speeches to increase the practicability of the fatigue prediction method, combined with the subsequent application scenarios. We used several common classifiers and LOO cross-validations, including LR, LDA, KNN, CART, NB, SVM, and MLP. By using the extracted audio features, we classified the recorded audio samples as normal and fatigued. The best average performance was achieved using the CART, with an accuracy of 76%, a recall of 81%, a precision of 76%, and an F1 of 76%. The detailed average results are shown in Figure 4.

## 4 Discussion

Fatigue is a state where physiological and psychological functions of the body are diminished. Mental fatigue has implications on decision-making ability and operational accuracy owing to the lack of concentration, which can significantly increase the risk of injury due to accidents (Williamson et al., 2011). Existing fatigue detection methods include subjective methods such as fatigue scales or employ professional instruments such as EEG/EMG, which are more demanding for operators and time-consuming. Moreover, they cannot detect the fatigue level in real time. Our fatigue detection method based on acoustic information can easily and rapidly determine the fatigue levels of the participants. This method can operate in real time and is non-invasive and efficient. Moreover, it can be combined with the advantages of information technology and big data to expand the applicability.

Currently, sleep deprivation modeling is a common method of fatigue modeling. Prolonged sleep deprivation affects human physiological rhythms and cognitive operations. This can subsequently increase the reaction time, decrease alertness and judgment, and significantly reduce operational effectiveness (Ma et al., 2020; Kerkamm et al., 2021). P300 latency can reflect the fatigue status in diseases such as multiple sclerosis (Chinnadurai et al., 2016), chronic fatigue syndrome (Dziadkowiak et al., 2015), and Parkinson’s disease (Pauletti et al., 2019). The results of this experiment show that 36 h of sleep deprivation increases the fatigue level of the participants. Moreover, it can change their physiological signals such as P300-related indicators and their salivary cortisol levels. Cortisol is a steroid hormone that belongs to the class of GCs and is produced mainly in the adrenal glands of the adrenal cortex in the zona fasciola and in lower amounts in other tissues of many animals. It is released in a diurnal cycle with an increased release under stress and low blood sugar concentrations. Cortisol functions by increasing blood sugar gluconeogenesis, suppressing the immune system and helping metabolize fats, proteins, and carbohydrates. Cortisol also reduces bone formation. Salivary cortisol level could reflect the health status. Studies have shown that GC level is closely related to a variety of unhealthy states, such as chronic fatigue syndrome (Herane-Vives et al., 2020), hearing loss (Bess et al., 2016; He et al., 2017; He et al., 2020), and stress (Fan et al., 2019; Pulopulos et al., 2020). It has been also used as a biomarker to reflect pathological-related statuses (Rubin et al., 2005; Vlenterie et al., 2016; Chang and Lin, 2017). In this experiment, we detected the level of GC in saliva to objectively reflect the fatigue degree of the participants, which provides the basis for our fatigue detection based on vocal information.

Speech contains substantial information that can be used as acoustic biomarkers to monitor patient status, diagnose conditions, classify diseases, or develop relevant drugs (Abrahamsson et al., 2018; Kraus, 2018; Noffs et al., 2018; Lee et al., 2020; Fagherazzi et al., 2021). Objective speech assessment is more accurate, replicable, and feasible than perceptual analysis (Noffs et al., 2018). Related studies have reported that changes in the characteristic parameters of the voice pattern are closely related to fatigue levels (de Vasconcelos et al., 2019). Our results also suggest that the corresponding parameters change with alterations in the fatigue level, and the characteristic parameters of the vocal pattern can characterize the fatigue state. This provides evidence for the rapid detection of the fatigue level of the body by vocal pattern information.

With the development of machine learning algorithms, artificial intelligence is widely used in speech information processing and applications (Dibeklioglu et al., 2018; Delić et al., 2019; Li and Mills, 2019). The machine learning approach intelligently matches speech features and physiological indicators without interfering with the work of the operator. This enables a person’s fatigue level to be rapidly and accurately identified through voice information (Dibeklioglu et al., 2018; de Vasconcelos et al., 2019). In our experiments, by using machine learning methods to verify the results of the data prediction of speech matching *via* physiological indicators, we showed that the accuracy rate lies within the range of 76%–81%. In our study, the number of participants was small. Consequently, the accuracies of the machine learning and identification approach were relatively low. However, the accuracy rate could be improved by increasing the sample size and optimizing the machine learning algorithm.

The use of voice information for fatigue determination is a relatively novel fatigue detection method, which can rapidly and non-invasively detect body fatigue. This method can address the limitations of existing detection methods, which are time-consuming, inconvenient, and cannot prevent accidents. However, this study has several limitations. For example, owing to the insufficient number of participants, females were not included in the experiment. In addition, different dialects and age groups were not considered. Due to the impact of the epidemic, it is difficult to carry out large-scale population experiments. We selected only male students from the same class as participants to reduce the impact of personal living habits, age, and sex; therefore, there would be some deviation in the sampling range. However, the main purpose of this experiment was to explore the methodology of fatigue detection based on vocal information. In the subsequent experiments, the sample size of voice data collection will be increased, and participants of different sexes and ages will be recruited for experiments to further update our speech database, optimize the evaluation algorithm, and then obtain a fatigue judgment method with a wider range of adaptability.

A “biological marker” or “biomarker” refers to medical signs that indicate the medical state observed from outside the patient (Strimbu and Tavel, 2010). Acoustic biomarkers have remarkable potential in reforming diagnostics in diseases affecting the heart, lungs, vocal folds, or brain, which can alter a person’s voice. Nowadays, speech recognition technology is one of the most promising technologies for improving healthcare services, and voice analyses *via* machine learning techniques provide new horizons in medicine. Studies on the biomarkers of the voice have been conducted in the field of neurodegenerative diseases, such as Parkinson’s disease, Alzheimer’s disease, and mild cognitive impairment (Ahmed et al., 2013; Dashtipour et al., 2018; Toth et al., 2018; Yang et al., 2020). Voice recognition has also been applied for the prediction and evaluation of health conditions, such as mental health and emotional condition (Pisanski et al., 2016; Holmqvist-Jämsén et al., 2017; Dibeklioglu et al., 2018; Pisanski and Sorokowski, 2021; Kappen et al., 2022). Recently, it has been applied to respiratory diseases such as COVID-19 to detect the health conditions of the patients and monitor the emotional states of the staff (Castillo-Allendes et al., 2021; König et al., 2021; Gama et al., 2022). However, no vocal biomarkers have been approved by the US Food and Drug Administration or the European Medicines Agency so far. In the future, a unified corpus collection standard and a large-scale library of clinically available voice samples should be developed, followed by algorithm optimization and updates and the integration of algorithms into user-friendly devices, such as smartphone applications and connected medical devices (Fagherazzi et al., 2021). With the integration and updates of big data, optimization of corresponding prediction algorithms, and continuous improvement of judgment accuracy and friendly interfaces, fatigue monitoring and early warning platforms based on acoustic information will achieve a real-time accurate fatigue judgment, as well as physiological, psychological, and pathology-related states, based on voices.

## Data Availability

The original contributions presented in the study are included in the article/supplementary material, further inquiries can be directed to the corresponding authors.
